# Superior mesenteric artery dissection with aneurysm, hematoma, and bowel ischemia mimicking pancreatitis: a rare case image report

**DOI:** 10.1093/omcr/omaf226

**Published:** 2025-11-26

**Authors:** Syed Rafay Hussain Zaidi, Muhammad Sheraz Hameed, Marwah Bintay Khalid, Usama Shafiq, Azka Asad Mirza, Muhammad Umer Javaid, Syed Saqib Ali Shah, Douglas Duffee, Rahmat Gul Omarzai, Muhammad Usama Naveed

**Affiliations:** Department of Medicine, Parkview Medical Center, Pubelo, United States; Department of Medicine, Rawalpindi Medical University, Rawalpindi, Pakistan; Department of Medicine, Rawalpindi Medical University, Rawalpindi, Pakistan; Department of Radiology, Shifa International Hospital, Islamabad, Pakistan; Department of Medicine, Al-Nafees Medical College, Islamabad, Pakistan; Department of Medicine, Federal Medical And Dental College, Islamabad, Pakistan; Department of Medicine, Rawalpindi Medical University, Rawalpindi, Pakistan; Department of Medicine, Parkview Medical Center, Pubelo, United States; Department of Medicine, Nangarhar medical University, Jalalabad, Afghanistan; Department of Medicine, Rawalpindi Medical University, Rawalpindi, Pakistan

**Keywords:** superior mesenteric artery, arterial dissection, Pseudoaneurysm, hematoma, tomography scanners, X-ray computed

## Abstract

Background: Superior mesenteric artery (SMA) dissection is an uncommon yet potentially fatal vascular condition that often manifests as acute abdominal pain complicates the diagnostic process. Contrast-enhanced CT is essential for diagnosis, revealing the extent of dissection and associated complications such as thrombosis or aneurysm. Case Presentation: We report a case of a 58-year-old male presenting with right hypochondriac and epigastric pain, accompanied by vomiting. Laboratory tests showed leukocytosis, elevated amylase and lipase, and a falling hemoglobin level, leading to a preliminary diagnosis of acute pancreatitis and initiation of supportive management. Persistent pain and progressive decline in hemoglobin prompted a contrast-enhanced CT, which revealed an isolated superior mesenteric artery dissection with a partially thrombosed false lumen, small pseudoaneurysmal dilation, and a significant intraperitoneal hematoma. Bowel wall thickening in the distal jejunum and proximal ileum suggested ischemia or early necrosis, with associated mild ascites and edematous bowel loops, but no pneumoperitoneum. Conclusion: Isolated SMA dissection with thrombosis, aneurysm, and hematoma can closely mimic acute pancreatitis, highlighting the importance of considering vascular causes in acute abdominal pain. Early use of contrast-enhanced CT guided by clinical red flags is crucial to prevent complications such as bowel necrosis. Our case demonstrates that, in hemodynamically stable patients, conservative management with supportive care, antihypertensives, and anticoagulation can result in favorable clinical and radiologic outcomes. This case demonstrates the integration of systematic CT imaging with clinical red-flag assessment to differentiate SMA dissections requiring urgent intervention from those amenable to conservative management, potentially reducing diagnostic delays and improving outcomes.

## Introduction

Acute abdominal pain presents a significant diagnostic challenge due to its wide range of causes and frequently inconclusive workups [1]. Diagnosis is further complicated by the need to consider vascular, visceral, parietal, referred, and psychogenic pain, particularly in elderly patients [2]. Among vascular causes, splanchnic artery dissection and mesenteric thrombosis are rare but potentially catastrophic, as they can rapidly progress to irreversible bowel injury.

While isolated arterial dissections are more commonly reported in carotid and renal arteries, visceral artery dissections are rare [3], with the superior mesenteric artery (SMA) most frequently affected. Isolated SMA dissection without aortic involvement is uncommon [4] and is presumed to result from an intimal or vasa vasorum tear, leading to hemorrhage within the medial and adventitial layers. SMA dissection typically presents as chronic abdominal pain worsened by meals, with potential hemodynamic instability from thrombus, luminal narrowing, or aneurysm.

Doppler ultrasound offers a non-invasive method for assessing blood flow but is operator-dependent and limited by patient factors. Computed Tomography Angiography (CTA) is preferred for definitive diagnosis due to rapid imaging and high resolution [5], though it carries radiation and contrast risks. MRA provides detailed imaging without radiation but is less suitable for acute diagnosis. This report describes an unusual case of SMA dissection with thrombosis and intraperitoneal hematoma, raising concern for bowel ischemia.

## Case presentation

### History of presenting illness

A 58-year-old male with a 20-year history of smoking, uncontrolled hypertension, and hypercholesterolemia presented to the emergency department with persistent severe abdominal pain in the right hypochondriac and epigastric regions. The pain, worsening over several days, was associated with nausea and intermittent vomiting, significantly affecting his appetite and daily activities. The patient denied abdominal trauma, bowel habit changes, or previous abdominal surgeries.

### Physical examination

On examination, his blood pressure was 160/100 mmHg, heart rate 92 bpm. Abdominal tenderness was present in the upper quadrants with mild guarding; bowel sounds were hypoactive, and no rebound tenderness was noted.

### Laboratory investigations

Tests revealed leukocytosis (WBC: 20960/μL), normal liver function, and mildly elevated pancreatic enzymes (lipase 108 U/L, amylase 180 U/L) [Table 1], initially suggesting acute pancreatitis. Additional findings included mild metabolic acidosis (bicarbonate 17 mEq/L), hyperglycemia (random glucose 199 mg/dL), and hypocalcemia (ionized calcium 4.2 mg/dL) [Table 1]. Lactate was normal, supporting against significant ischemia. Enzyme levels did not meet the >3x ULN threshold for pancreatitis. Supportive management was initiated, but persistent pain and progressive hemoglobin decline raised concerns about ongoing blood loss.

**Table 1:** Laboratory investigations—Complete blood count and biochemistry.

Parameter	Results	Units	Reference range
Hematology			
WBC TOTAL	20 960	/μl	(4000/μl – 11 000/μl)
RBC, Total	4.45	m/μl	M (4.5–6.5) m/μl F (3.8–5.8) m/μl
Hemoglobin	9.2	g/dl	M (13.0–18.0) g/dl F (11.6–16.5) g/dl
HCT	29.3	%	M (40–50) % F (38–47) %
MCV	65.8	fL	(80–90) fL
MCH	20.7	pg	(27–32) pg
MCHC	31.4	g/dl	(33–38) g/dl
Platelet count	204 000	/μl	(150000-400 000)/μl
Neutrophils	82	%	(40–75) %
Lymphocytes	7	%	(20–45) %
Biochemistry			
Lipase serum	108	U/L	(13–60) U/L
Amylase	180	U/L	(31–107) U/L
Lactate	2.0	mmol/L	(05–2.2) mmol/L
CRP	4.1	mg/L	<5 mg/L
ESR	14	mm/hr	M: (0–15) mm/hr F: (0–20) mm/hr
LDH	232	U/L	140–280 U/L
PT	13	Sec	11–14 sec

### Radiological imaging

**Figure 1:**
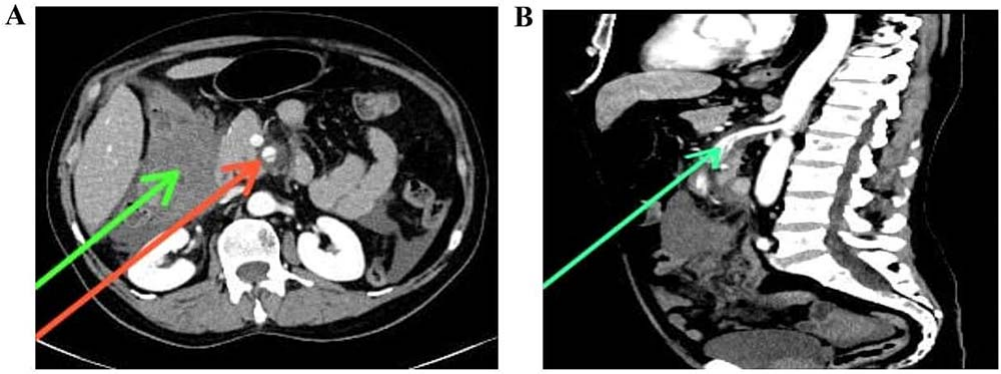
(A) CT aortogram (axial view at the level of the kidneys) showing an intimal flap dissecting the SMA (red arrow) and hemoperitoneum in the right hypochondriac region (green arrow). (B) CT aortogram (sagittal view, arterial phase) demonstrating a focal intimal flap dissecting the SMA.

**Figure 2:**
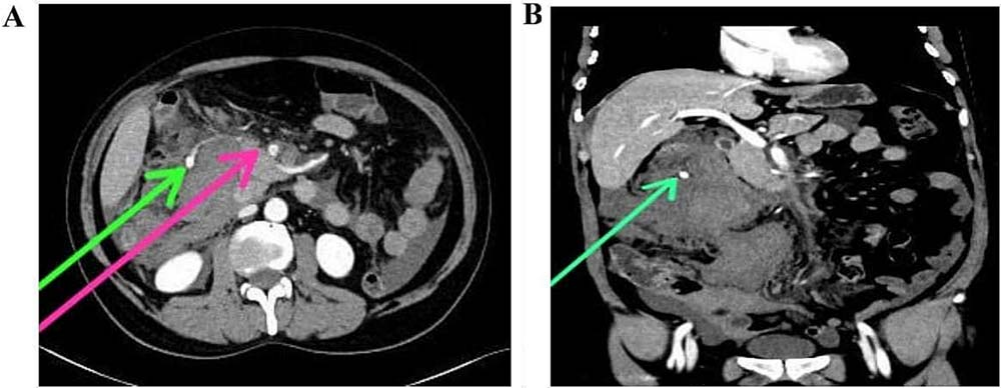
(A) Tiny branch originating from the SMA extending into the right side of the peritoneal cavity (pink arrow). A terminal outpouching/aneurysm (green arrow) shows contrast extravasation, suggesting the source of active arterial bleeding. (B) CT aortogram (coronal view, arterial phase) again demonstrates the aneurysm (originating from a side branch of the SMA) in the right hypochondrium, surrounded by hyperdense fluid/hemoperitoneum.

Abdominal ultrasound ruled out cholecystitis, prompting a contrast-enhanced CT scan, which revealed a 4 cm segmental dissection of the proximal superior mesenteric artery (SMA) with a partially thrombosed false lumen [Fig. 1A and B]. A small pseudoaneurysmal dilation (1.5 cm) was noted [Fig. 2A], with surrounding fat stranding and edema [Fig. 2B]. A large intraperitoneal hematoma (9.6 × 11.7 × 10 cm) contributed to hemoglobin decline. Bowel wall thickening in the distal jejunum and proximal ileum suggested ischemia, with mild ascites and no pneumoperitoneum.

### Diagnosis

Findings confirmed isolated SMA dissection with thrombosis, pseudoaneurysmal formation, intraperitoneal hematoma, and possible ischemic bowel injury, revising the initial diagnosis of pancreatitis.

### Treatment

Given hemodynamic stability, conservative management was continued with bowel rest, antihypertensives, and anticoagulation. Serial abdominal exams and hemoglobin monitoring were performed. Symptoms gradually improved, hemoglobin stabilized, and repeat imaging showed no progression of dissection, with a reducing hematoma. The patient was discharged after six days with outpatient vascular surgery follow-up, continued anticoagulation targeting INR 2–3, and optimized antihypertensive therapy.

## Discussion

Isolated superior mesenteric artery dissection (ISMAD) is a rare emergency, often incidentally diagnosed in patients presenting with acute abdominal pain, nausea, vomiting, or diarrhea [6]. First described in 1947, ISMAD is increasingly recognized due to advancements in imaging, particularly contrast-enhanced CT, CT angiography (CTA), and magnetic resonance angiography (MRA). Despite this, the condition remains uncommon and is frequently misdiagnosed because its nonspecific presentation can mimic more prevalent gastrointestinal conditions, such as pancreatitis, as in this case.

Several classification systems for ISMAD exist, though no consensus has been reached [7]. The Sakamoto classification categorizes dissections by false lumen appearance [8], whereas Yun’s classification uses angiographic findings, considering false lumen flow and true lumen patency. Sakamoto’s system is more useful for surgical or radiologic monitoring, while Yun’s framework aids multidisciplinary planning and guides the balance between conservative management and intervention.

Our patient, a 58-year-old male with poorly controlled hypertension and long-term smoking, presented with acute-onset upper abdominal pain caused by SMA dissection complicated by thrombosis, pseudoaneurysmal dilation, intraperitoneal hematoma, and bowel ischemia. Risk factors for ISMAD include hypertension, atherosclerosis, connective tissue disorders, and fibromuscular dysplasia [9]. The pathophysiology involves intimal or vasa vasorum tears leading to false lumen formation, which may progress to thrombosis, pseudoaneurysm, or rupture. Elevated pancreatic enzymes in this patient likely reflected ischemia rather than primary pancreatitis, emphasizing the importance of early imaging.

Management depends on symptom severity, hemodynamic stability, and complications such as bowel ischemia or rupture. Conservative therapy—including bowel rest, blood pressure control, and anticoagulation—is favored in stable patients without infarction or peritonitis [10]. Our patient responded well, avoiding surgical or endovascular intervention. Intervention is indicated for persistent bleeding, bowel necrosis, or progressive pseudoaneurysm. Stenting has shown favorable outcomes in select cases, though long-term data are limited [10]. Close follow-up with imaging is essential to detect delayed complications such as re-dissection or thromboembolic events.

This case highlights SMA dissection mimicking pancreatitis and demonstrates the value of integrating an imaging-based severity score with a clinical red-flag checklist. This approach helps differentiate cases requiring urgent intervention from those suitable for conservative management, potentially reducing bowel loss and mortality. Incorporating this protocol into emergency and gastroenterology pathways may prevent delays in the diagnosis and management of high-risk vascular abdominal pain.

## Conclusion

ISMAD complicated by thrombosis, aneurysm, hematoma, and suspected bowel ischemia is a rare and complex cause of abdominal pain. This presentation underscores the importance of considering vascular etiologies in the differential diagnosis of acute abdominal pain, particularly in high-risk patients. Prompt imaging such as CTA or MRA along with appropriate risk stratification, is crucial. Management options include conservative therapy, anticoagulation, endovascular stenting, or open surgical repair, with the choice guided by clinical status and complication risk.

## Data Availability

Data will be provided on request by corresponding author.
